# Lactylation-driven METTL3 regulates wound healing by enhancing m6A/HNRNPA2B1/DNMT1 signaling in keratinocytes

**DOI:** 10.1016/j.gendis.2025.101787

**Published:** 2025-07-28

**Authors:** Xue-ting Hu, Lu-min Sui, Cheng-xiu Pu, Luo-quan Ao, Mu Yuan, Li Deng, Qing Zhao, Xiao-feng Wu, Xiang Xu

**Affiliations:** a Department of Stem Cell and Regenerative Medicine, State Key Laboratory of Trauma and Chemical Poisoning, Daping Hospital, Army Medical University, Chongqing 400042, China; b Yunnan Key Laboratroy of Stem Cell, Regenerative Medicine & School of Rehabilitation, Kunming Medical University, Kunming, Yunnan 650500, China; c Skin and Medical Aesthetics Center of the Second Affiliated Hospital of Chongqing Medical University, Chongqing 400010, China

Chronic non-healing wounds, such as diabetic foot ulcers, represent a clinical challenge with an increasing incidence.^1^ A deeper understanding of the molecular mechanisms underlying wound healing is of great significance. RNA m6A modification is a widespread and important epigenetic regulatory mechanism,^2^ but its role in wound healing remains unclear. In this study, we found that METTL3, an RNA m6A methyltransferase, is a positive regulator of wound healing and that its low expression is closely related to chronic diabetic wounds. Mechanistically, we found that METTL3 regulates m6A modification of DNMT1 mRNA, increasing its expression and promoting keratinocyte proliferation. Additionally, we found that HNRNPA2B1 is the m6A “reader” that assists in regulating DNMT1 expression; HNRNPA2B1 can recognize m6A modifications on DNMT1 mRNA and increase its stability. Moreover, we discovered that lactate in the wound microenvironment accounts for the up-regulation of METTL3 expression during wound healing by inducing histone H3K18 lactylation. Finally, using a mouse wound model, we found that applying lactate or the lactylation “eraser” inhibitor LBH589 to the wound site promoted wound healing by up-regulating METTL3 expression. Our findings provide new targets for chronic non-healing wounds and suggest that lactylation modulation could serve as a potential therapeutic strategy for accelerating wound healing.

We examined the changes in the expression of m6A regulators (METTL3, METTL14, WTAP, FTO and ALKBH5) during wound healing (D1, D3, D5, D7, D9). The qPCR results revealed that METTL3 was the only gene exhibiting a progressively increasing trend in expression (Fig. 1A). Western blot analysis and immunohistochemical staining confirmed that METTL3 expression increased during wound healing (Fig. 1B and C). Additionally, compared with that in normal mouse skin tissue, METTL3 expression was lower in the skin tissue of diabetic mice (Fig. S1A and S1B), and METTL3 expression did not increase with the progression of wound healing in diabetic mice (Fig. S1C). Furthermore, when we injected siRNAs targeting METTL3 or the METTL3 inhibitor STM2457 into the wound sites of the mice, we found that wound healing was significantly delayed (Fig. 1D and E; Fig. S1D–S1H). These results indicate that METTL3 is a positive regulator of wound healing.

**Figure 1:**
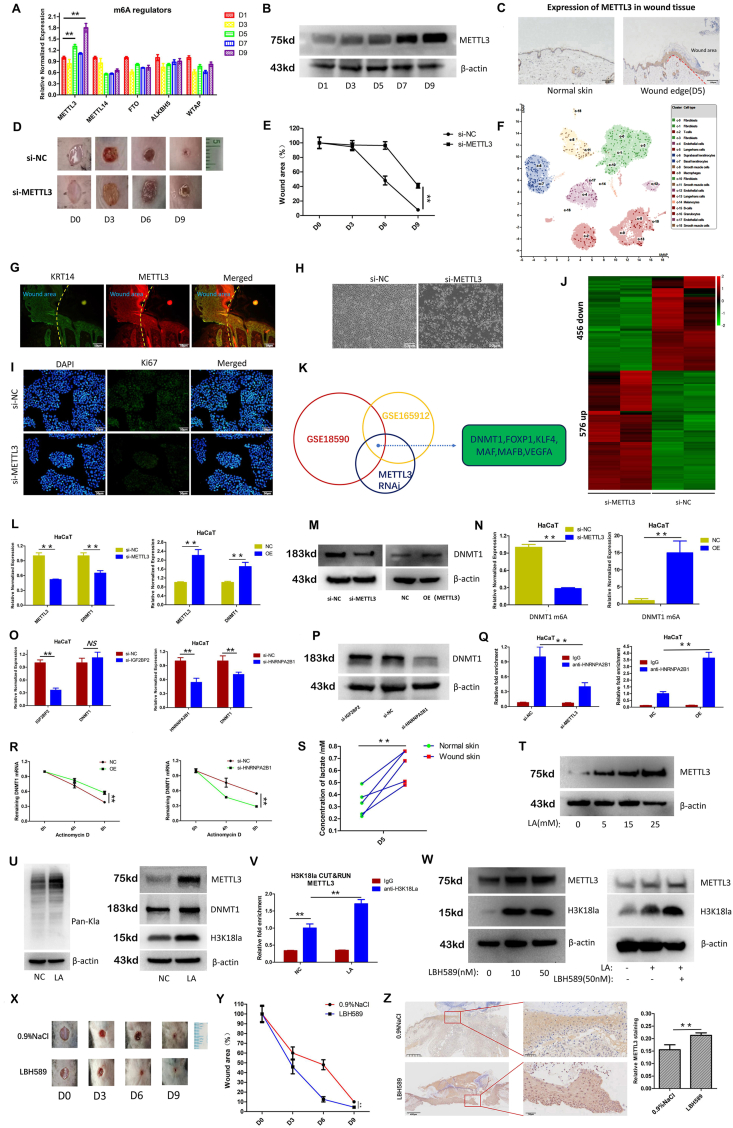
Lactylation-driven METTL3 promotes wound healing. **(A)** Changes in the expression of RNA m6A regulators during the wound healing process detected by qPCR. **(B)** Changes in METTL3 expression during the wound healing process detected by Western blot (WB). **(C)** Immunohistochemical staining for METTL3 expression at the wound site (Day 5). **(D, E)** Effects (D) and quantification (E) of METTL3 siRNAs on mouse wound healing (*n* = 5). The siRNA (50 nM) was injected into the wound site of the mice every three days. **(F)** Single-cell sequencing analysis was performed to examine METTL3 expression in various types of skin cells. Data were collected from the HPA database. **(G)** Immunofluorescence staining for METTL3 expression in wound tissue (D5). **(H)** Effects of METTL3 siRNAs on the growth of HaCaT keratinocytes. **(I)** Effects of METTL3 siRNAs on the proliferation of HaCaT keratinocytes detected by Ki-67 staining. **(J)** Effects of METTL3 interference on gene expression in HaCaT keratinocytes detected by whole-transcriptome sequencing. **(K)** Integration analysis of differentially expressed genes with genes involved in keratinocyte proliferation changes (GSE18590) and m6A changes during proliferation processes (GSE165912). **(L, M)** Effects of METTL3 interference or overexpression on DNMT1 expression detected by qPCR (L) and WB (M). **(N)** Effects of METTL3 interference and overexpression on m6A levels in DNMT1 mRNA analyzed by MeRIP experiments. **(O, P)** Effects of interfering with IGF2BP2 and HNRNPA2B1 on DNMT1 expression in HaCaT cells detected by qPCR (O) and WB (P). **(Q)** The impact of interfering with or overexpressing METTL3 on the interaction between the HNRNPA2B1 protein and DNMT1 mRNA detected by RNA Immunoprecipitation (RIP). **(R)** Effects of METTL3 and HNRNPA2B1 on DNMT1 mRNA stability. METTL3-overexpressing or si-HNRNPA2B1 keratinocytes were treated with 5 μg/mL actinomycin D. **(S)** Measurement of the lactate content in the wound microenvironment (Day 5). **(T)** Effects of lactate treatment on METTL3 expression detected by WB. **(U)** Effects of lactate stimulation on H3K18la and DNMT1 expression downstream of METTL3 detected by WB. **(V)** Effects of lactate treatment on the binding of H3K18la to the METTL3 promoter detected by the CUT&RUN assay. **(W)** Effects of the HDAC inhibitor LBH589 alone or in combination with lactate on H3K18la and METTL3 expression detected by WB. **(X, Y)** Effects (×) and quantification (Y) of the HDAC inhibitor LBH589 on wound healing in mice (*n* = 5). LBH589 (50 nM) was injected into the wound site of the mice every three days. **(Z)** Effects of LBH589 on METTL3 expression in the wound tissue of mice detected by immunohistochemistry (IHC). ∗∗*p* < 0.01; ∗*p* < 0.05, NS: no significance.

Our previous immunohistochemical staining results showed that METTL3 is expressed primarily in the epidermal layer of the skin (Fig. 1C). Additionally, by querying single-cell data from the Human Protein Atlas database, we found that METTL3 is indeed expressed mainly within keratinocytes in skin tissue (Fig. 1F), and immunofluorescence staining revealed that METTL3 is expressed predominantly in keratinocytes (Fig. 1G). Therefore, we speculate that METTL3 functions by influencing the function of keratinocytes. By interfering with METTL3 or overexpressing METTL3 in keratinocytes, as well as utilizing the METTL3 inhibitor STM2457, we found that METTL3 had no effect on keratinocyte migration but promoted its proliferation (Fig. 1H and I; Fig. S2A–S2O). These results indicate that METTL3 regulates wound healing by promoting keratinocyte proliferation.

We used RNA-seq to explore the downstream target genes of METTL3. The results showed that silencing METTL3 in keratinocytes led to decreased expression of 456 genes and increased expression of 576 genes (Fig. 1J). Moreover, Kyoto Encyclopedia of Genes and Genomes (KEGG), Gene Ontology (GO) and Gene Set Enrichment Analysis (GESA) analyses indicated that METTL3 deficiency in keratinocytes affected cell proliferation (Fig. S3A–S3C). By integrating differentially expressed genes (GSE18590) and genes with altered m6A modification levels (GSE165912) under conditions of altered keratinocyte proliferation, we identified six potential downstream target genes of METTL3 (Fig. 1K). Among these candidate genes, DNMT1 is an important regulator of keratinocyte proliferation (Fig. S3D and S3E).^3^ Besides, we found that the expression of DNMT1 gradually increased during the wound healing process, similar to that of METTL3 (Fig. S3F and S3G). In addition, we detected a positive correlation between METTL3 and DNMT1 expression in skin tissues (Fig. S3H). Therefore, we decided to focus on it. Verification by qPCR and Western blot revealed that silencing METTL3 in keratinocytes indeed reduces DNMT1 expression, whereas overexpressing METTL3 increases its expression (Fig. 1L, M). Furthermore, we identified multiple m6A modification sites within the DNMT1 mRNA sequence (Fig. S3I and S3J). Through m6A dot blot assays and MeRIP-qPCR analysis, we found that silencing and overexpressing METTL3 inhibited and up-regulated, respectively, both the overall RNA m6A modification level and the m6A modification level of DNMT1 mRNA (Fig. 1N; Fig. S3K). Additionally, treating keratinocytes with the METTL3 inhibitor STM2457 also reduced DNMT1 expression and its mRNA m6A modification levels (Fig. S3L–S3N). These results suggest that DNMT1 is the target gene of METTL3-mediated RNA m6A modification in regulating keratinocyte proliferation.

We attempted to identify the m6A “readers” that regulate DNMT1 expression. By analyzing genes that are differentially expressed during keratinocyte proliferation,^3^ we shortlisted the following m6A “readers”: YTHDC1, IGF2BP2, HNRNPA2B1, EIF3H, and EIF3B (Fig. S4A). Furthermore, by querying the GTEx database, we found that HNRNPA2B1 and IGF2BP2 expression was positively correlated with DNMT1 expression in skin tissue, especially HNRNPA2B1 expression (Fig. S4B and S4C). When we transfected siRNAs targeting HNRNPA2B1 and IGF2BP2 into keratinocytes, the results indicated that silencing HNRNPA2B1 significantly reduced DNMT1 expression, whereas IGF2BP2 had no such effect (Fig. 1O and P). Moreover, RNA Immunoprecipitation (RIP) assays revealed that HNRNPA2B1 directly binds to DNMT1 mRNA and that their interaction decreases significantly after silencing METTL3 and increases upon overexpression of METTL3 (Fig. 1Q). Besides, RNA stability assays showed that both HNRNPA2B1 and METTL3 enhance the stability of DNMT1 mRNA (Fig. 1R). Additionally, we found that silencing HNRNPA2B1 significantly inhibited keratinocyte proliferation (Fig. S4D–S4I). These findings indicate that HNRNPA2B1 is the m6A “reader” that regulates the stability of DNMT1 mRNA and the proliferation of keratinocytes.

Elevated lactate levels are a prominent characteristic of the wound microenvironment. Our results showed that lactate levels were indeed significantly elevated at the wound site compared with those in normal skin tissue (Fig. 1S) and that lactate promoted METTL3 expression in a dose-dependent manner (Fig. 1T). Recent studies have found that lactate can regulate gene expression by inducing histone lactylation.^4^ The results showed that lactate treatment indeed significantly increased the pan-Kla and H3K18la levels in keratinocytes (Fig. 1U) and induced METTL3 and DNMT1 expression as well as m6A modification (Fig. S5A and S5B). Besides, when endogenous lactate production was inhibited by 2-DG, the expression of METTL3, its downstream target DNMT1, and H3K18la was all markedly down-regulated in keratinocytes. Conversely, when keratinocytes were treated with rotenone, an inhibitor of cellular aerobic respiration, there was a noticeable increase in METTL3, DNMT1, and H3K18la (Fig. S5C–S5E). These results suggest that lactate induces H3K18la and up-regulates the expression of METTL3 and DNMT1 in keratinocytes. Furthermore, through CUT&RUN experiments, we found that lactate stimulation induced H3K18la enrichment at the METTL3 promoter region (Fig. 1V) but not at the promoter region of DNMT1 (Fig. S5F). Histone deacetylases (HDACs) 1–3 have been identified as histone lysine delactylases.^5^ When we treated keratinocytes with the pan-HDAC inhibitor panobinostat (LBH589), we found H3K18la and METTL3 expression increased significantly (Fig. 1W). Additionally, we found that lactate treatment accelerated keratinocyte proliferation (Fig. S5G–S5L). These results indicate that lactate accumulation in the wound microenvironment is sufficient to activate METTL3 transcription and keratinocyte proliferation by inducing H3K18la.

Finally, we established a mouse wound model and applied lactate or the HDAC inhibitor LBH589 to the wound sites. Compared with the saline treatment, the application of lactate or LBH589 significantly accelerated wound healing in mice (Fig. 1X, Y; Fig. S6A–S6D, S6G, S6H). Additionally, immunohistochemical staining demonstrated that treatment with lactate or LBH589 significantly increased METTL3 expression at wound sites (Fig, 1Z; Fig. S6E and S6F). These results indicate that modulating lactylation with a lactate or HDAC inhibitor can indeed promote METTL3 expression and enhance wound healing *in vivo*.

In this study, we uncovered the novel role of lactylation-driven METTL3 in regulating wound healing (Fig. S6I), offering new targets and therapeutic strategies for chronic non-healing wounds.

## CRediT authorship contribution statement

**Xue-ting Hu:** Writing – original draft, Visualization, Project administration, Methodology, Funding acquisition, Conceptualization. **Lu-min Sui:** Methodology, Investigation. **Cheng-xiu Pu:** Methodology, Investigation. **Luo-quan Ao:** Methodology, Investigation. **Mu Yuan:** Methodology, Investigation. **Li Deng:** Methodology, Investigation. **Qing Zhao:** Methodology, Investigation. **Xiao-feng Wu:** Project administration, Methodology, Investigation, Data curation. **Xiang Xu:** Writing – review & editing, Funding acquisition.

## Ethics declaration

This study received approval from the Ethics Committee on Animal Experiments of Army Medical University (Approval No. AMUWEC20237012). All animal experiments were carried out in accordance with the guidelines set forth by international committees for the care and use of laboratory animals.

## Data availability

The datasets used in this study are available from the corresponding author upon reasonable request.

## Funding

This research was funded by the 10.13039/501100002858China Postdoctoral Science Foundation (No. 2024M754253), the 10.13039/501100001809National Natural Science Foundation of China (No. 82372529), the 10.13039/100012546Chongqing Postdoctoral Science Foundation (No. CSTB2023NSCQ-BHX0015), the Science and Technology Innovation Enhancement Project of the Army Medical University (China) (No. 2022XQN45), Postdoctoral Foundation of Daping Hospital (China) (No. ZXBSH004) and Chongqing Key Laboratory of Precision Diagnosis and Treatment for Kidney Diseases (China).

## Conflict of interests

The authors declare that they have no competing interests.
