# Supplementary material for: Lactylation-driven METTL3 regulates wound healing by enhancing m6A/HNRNPA2B1/DNMT1 signaling in keratinocytes
Source: Genes Dis. 2025 Jul 28;13(3):101787. doi: 10.1016/j.gendis.2025.101787 (PMC12856603; doi:10.1016/j.gendis.2025.101787)
Supplement: Multimedia component 1 [file mmc1.doc]

**Supplementary information**

**Supplementary materials and methods**

**Keratinocyte isolation and culture**

Primary skin keratinocytes were obtained from 1 to 2-day-old mice, and the extraction protocol followed the protocol of Li et al. [1]; the protocols were approved by the Ethics Committee of Army Medical University. The main steps were as follows: newborn mice were disinfected with 75% ethanol and then placed on a laminar flow hood. Their limbs were removed with scissors, and the skin was peeled off via forceps. The skin was then washed and placed in a medium containing 4 mg/mL dispase II (Solarbio, D6430, China) overnight at 4°C. After that, the digested skin tissue was removed, and the epidermal layer was gently stripped off using forceps and placed in 0.25% trypsin for further digestion at room temperature for 20 minutes. Finally, the isolated keratinocytes were filtered, centrifuged, and resuspended before being plated into the culture medium. Primary mouse keratinocytes were maintained in KC basal medium (EpiLife, MEPICF500, Invitrogen, Carlsbad, CA), which included 0.06 mM calcium chloride and was further enriched with defined growth supplement (dGS, product code S0125, also from Invitrogen, Carlsbad, CA). The human immortal keratinocyte line HaCaT cells were obtained from ATCC and cultured in DMEM supplemented with 10% FBS. All the cells were cultured at 37˚C with 5% CO_2_ in a humidified incubator. METTL3 inhibitor STM2457(MCE, HY-134836, USA), DNMT1 inhibitor GSK-3484862(MCE, HY-135146, USA), and pan-HDAC inhibitor Panobinostat (MCE, HY-10224, USA) was added to the culture medium to investigate the function of the relevant molecule(s).

**RNA isolation and m6A dot blot**

Total RNA was extracted from skin wound tissues or cells using TRIzol reagent via the following steps. First, crushed tissues or cells were lysed with 1ml of TRIzol for 5 minutes at room temperature. After that, 200µl of chloroform was added to the system, which was subsequently vortexed for 15 seconds. After centrifugation at 4°C, and 12000 rpm for 10 minutes, the supernatant was transferred to a new centrifuge tube, and an equal volume of isopropanol was added to the tube. The samples were subsequently mixed in a centrifuge tube and then centrifuged at 4°C, and 12000 rpm for 10 minutes. Finally, the RNA pellet was washed with 75% ethanol and dissolved in ddH_2_O.

For the RNA m6A dot blot assay, total RNA was heated to 65℃ for 5 mins and added to a nitrocellulose membrane. After being crosslinked by ultraviolet (UV) light for 30 min, the membrane was blocked with 5% nonfat milk for 1 h and then incubated with an anti-m6A antibody (1:1000, Beyotime, AF7407) at 4 °C overnight. After that, the blots were washed and then incubated with HRP-conjugated goat anti-rabbit IgG (CST, 7074, 1:5000) for 2 h. Finally, the proteins in the membrane were detected with a chemiluminescence detection system (Bio-Rad, USA).

**qRT-PCR**

Total RNA was extracted, and then, the genomic DNA was removed via a reverse transcription kit, followed by reverse transcription of the RNA into cDNA(Vazyme, R223-01,China). Real-time PCR was performed as previously described[2]. The gene-specific primers used for qRT-PCR used in this study are shown below (Table S1). The relative expression of the RNAs was calculated via the comparative Ct method and β-actin mRNA was used as an endogenous control to normalize for differences in the amount of total RNA.

**Protein extraction and Western blot**

Total protein was extracted from skin wound tissues or cells using RIPA lysis buffer (Beyotime, P0013C, China) with the following steps. Crushed tissues or cells were lysed with RIPA lysis buffer on ice for 30 minutes. After centrifuging at 4°C, 12000 RPM for 10 minutes, the supernatant was collected, and 5x SDS loading buffer was added. The mixture was subsequently boiled for 10 minutes. The prepared protein samples were stored at -20°C.

Western blotting was performed as previously described[3]. Briefly, total protein was separated on a 10-12% SDS-PAGE gel and then transferred to a PVDF membrane (0.45 μm, Millipore). After that, the membrane was blocked with 5% nonfat milk for 1 h and then incubated with primary antibodies specific to METTL3 (Abcam, ab195352,1:1000), Ki-67 (Abcam, ab16667,1:1000), KRT14 (Abcam, ab181595,1:1000), DNMT1 (ABclonal, A22455,1:1000), HNRNPA2B1 (Santa Cruz, sc-374053,1:500), Pan-kla (PTM bio, PTM-1401RM,1:1000), H3K18la (PTM bio, PTM-10406RM ,1:1000) and β-Actin (CST, 3700,1:1000) overnight at 4℃. After that, the membranes were washed and then incubated with goat anti-rabbit IgG(H+L) HRP (Multi Sciences, GAR007, 1:5000) or goat anti-mouse IgG(H+L) HRP (Multi Sciences, GAM007, 1:5000) for 2 h. Finally, the proteins in the membranes were detected via a chemiluminescence detection system (Bio-Rad, USA).

**H&E staining and immunochemistry**

For H&E staining, seven-micrometer-thick skin wound sections were deparaffinized and rehydrated, incubated with Harris’s hematoxylin and eosin Y, dehydrated in graded alcohols (70%, 95%, 100%) and cleared with xylene for 5 minutes. Finally, the slides were sealed with neutral resin and observed under a microscope.

For immunochemistry, after being dewaxed and hydrated, the sections were subjected to antigen retrieval via citric acid buffer via the microwave repair technique, and were subsequently blocked with PBS containing 10% bovine calf serum for 1 hour at room temperature. After that, the METTL3 antibody (Abcam, ab195352,1:200) was diluted in PBS with 1% BSA and incubated with the sections overnight at 4℃, and SignalStain® Boost Detection Reagent (CST, HRP, 8114) was used as the secondary antibody. Finally, the slides were examined with a tissue slice scanning system (KFBIO, KF-PRO-005-EX, China).

**Single-cell sequencing analysis of METTL3 expression in skin cells**

The METTL3 expression single-cell data originate from the HPA database (https://www.proteinatlas.org). The search keywords were, in sequence: METTL3, single cell, and skin.

**siRNAs and gene transfection**

Pooled or single siRNAs targeting METTL3, HNRNPA2B1 and IGF2BP2 were synthesized and purchased from RiboBio Company (Guangzhou, China). The sequence of siRNAs used in this study are shown below (Table S2). The siRNAs were transfected into keratinocytes via the Lipofectamine™ RNAi MAX transfection reagent (Life, 13778150, USA) following protocols recommended by the manufacturer. The cells were harvested 48 h post-transfection for further investigations.

**Immunofluorescence**

Keratinocytes were cultured on coverslips before use. The coverslips were fixed with 4% paraformaldehyde for 10 min and then blocked with 5% BSA at room temperature for 1 h. Primary Ki-67 antibody (Abcam, ab16667, 1:100) was diluted in PBS with 1% BSA and incubated with the coverslips overnight. After that, the coverslips were washed and incubated with goat anti-rabbit IgG (Alexa Fluor 488, CST, 4412,1:500) for 2 h and the nuclei was stained with 0.1 mg/l DAPI in PBS for 1 minute. Finally, the coverslips were examined with a fluorescence microscope (Olympus, Japan).

As for the fluorescent double staining of KRT14 and METTL3, we took the following steps. The coverslips were fixed and blocked as before. After that, the coverslips were incubated with KRT14 Mouse mAb (CST, 48020,1:100) and METTL3 Rabbit mAb (Abcam, ab195352,1:100) overnight. And then, the coverslips were washed and incubated with goat anti-mouse IgG (Alexa Fluor 488, CST, 89853,1:500) and goat anti-rabbit IgG (Alexa Fluor 647, CST, 4414,1:500) for 2 h. Finally, the coverslips were examined with a fluorescence microscope (Olympus, Japan).

**EdU Assay**

The EdU assay was performed using a Cell-Light^TM^ EdU Apollo In Vitro Kit (Ribo Bio, C10310, China) following the protocols recommended by the manufacturer. Briefly, EdU was added to the keratinocyte culture medium, and the mixture was incubated with the cells for 2 hours, followed by fixation with 4% paraformaldehyde at room temperature for 30 minutes. After that, the cells were washed and incubated with 1X Apollo® staining reaction solution for 30 minutes. The nuclei were stained with 0.1 mg/l DAPI in PBS for 1 minute. Finally, the cells were examined with a fluorescence microscope (Olympus, Japan).

**METTL3 gene overexpression**

Lentiviruses for METTL3 overexpression were packaged by Shanghai Jikai Gene Chemical Technology Co. Ltd. Human METTL3 ((NM_019852)) was chemically synthesized and cloned into the lentiviral expression vector GV358. Keratinocytes infected with lentivirus (MOI:20) were continuously selected for one week in a culture medium containing 2 µg/ml puromycin, ensuring that the percentage of positive cells was greater than 95%.

**Full transcriptome RNA sequencing**

Total RNA was extracted from si-NC and si-METTL3 transfected keratinocytes as described above. Alteration in gene expression profiles were examined via RNA sequencing at LC Bio (Zhejiang, China). The differentially expressed genes were determined with a threshold cutoff of 2-fold (p < 0.05).

**Gene expression correlation analysis**

The correlations among METTL3, DNMT1, HNRNPA2B1 and IGF2BP2 expression levels in skin tissue were assessed via the Gene Expression Profiling Interactive Analysis (GEPIA, http://gepia.cancer-pku.cn/detail.php), an innovative online tool designed for exploring RNA sequencing data. This tool provides analysis of data from 9,736 tumor samples and 8,587 normal samples sourced from The Cancer Genome Atlas (TCGA) and the Genotype-Tissue Expression (GTEx) projects[4]. The Pearson correlation coefficient was used for statistical analysis.

**MeRIP-qPCR**

Changes in the m6A modification levels of DNMT1 mRNA were detected by a ribo*MeRIP*^TM^ m6A Transcriptome Profiling Kit (Ribo Bio, C11051, China) following protocols recommended by the manufacturer. Briefly, total RNA was fragmented into 100-150 nt pieces via RNA fragmentation buffer and repurified before use. After that, the fragmented RNA was incubated with 1X IP buffer containing magnetic beads A/G conjugated with the m6A antibody at 4℃ for 2 h. Finally, the RNA was eluted from the magnetic beads and detected by qPCR. The primers for DNMT1 MeRIP-qPCR used in this study are shown below:

Part ① Forward：AGCAACGGGCAGATG；Reverse：ACAGCTCCAGAGGGA

Part ② Forward：CGTTCCGGCTGAACA；Reverse：GTGGTCTCCCCAGCC

Part ③ Forward：TGGCTGCCCGCATGC；Reverse：CCGTCTGAGAGCCGC

**RNA-binding protein immunoprecipitation**

An anti-HNRNPA2B1 RIP assay was performed to detect the interaction between HNRNPA2B1 and DNMT1 mRNA via a BersinBio^TM^ RNA Immunoprecipitation (RIP) Kit (BersinBio, Bes5101, China) following the manufacturer’s instructions. In brief, the keratinocytes were rinsed with cold PBS and subsequently lysed with RIP lysis buffer. Next, the cell lysates were incubated overnight with protein A/G magnetic beads that had been coated with an HNRNPA2B1 antibody (Santa Cruz, sc-374053, 1:100). Normal mouse IgG was used as a negative control. Finally, RNAs that bind to HNRNPA2B1 were extracted with TRIzol reagent (Sigma, USA) and detected via qRT-PCR.

**RNA stability assay**

For analysis of DNMT1 mRNA stability, METTL3 overexpressing or si-HNRNPA2B1 keratinocytes were treated with 5 μg/ml actinomycin D (Selleck, S8964, USA). The cells were harvested at 0, 4, and 8-hour time points, and total RNA was isolated using TRIzol reagent (Sigma, USA). Reverse transcription was carried out as previously described, and the mRNA levels were quantified via qRT-PCR.

**Measurement of lactate content in mouse tissue**

The lactate content in normal skin and wound tissues of mice was measured using the Beyotime Lactate Assay Kit (Beyotime, S0208S, China) and the operations were carried out according to the kit's instructions. In brief, use scissors to excise skin tissues from both the normal and wound areas of mice. Then, homogenize the tissues and centrifuge them. Next, add the supernatant and standard solutions separately to the working solution for the reaction. After that, use a spectrophotometer to measure the optical density (OD) values. Finally, calculate the lactate content of the samples based on the standard curve.

**Cleavage under targets and release using nuclease (CUT&RUN) and qPCR**

The CUT&RUN assay[5] was used to detect the interaction between H3K18la and the METTL3 promoter. The experiment was conducted using the CUT&RUN Assay Kit (CST, 86652, USA) in accordance with the manufacturer’s protocol. DNA fragments associated with H3K18la were isolated through phenol/chloroform extraction and subsequently analyzed by qPCR using primers that target the promoter regions of the METTL3 gene. The PCR primers used were as follows: forward, GCGCCTTATTCGAGAGGTGT; reverse, CTTGTGGGCCTGGATAGAGC.

**Animal study**

All animal experiments were approved by the Ethics Committee on Animal Experiments of Army Medical University (AMUWEC20237012). C57BL/6 and db/db mice were purchased from Sibeifu (Beijing) Biotechnology Co., Ltd.

The method for establishing a mouse skin wound model followed previously described procedures [6]. Briefly, eight-week-old mice were anesthetized, and their back fur was removed; then, circular skin areas with a diameter of 8 mm were excised using scissors to prepare the wound model. The medication(40μl of 20μM siRNAs or 10µM STM2457 or 25mM lactate or 50nM LBH589) was applied to the wound area via a syringe every three days. The wound healing process was observed and photographed every three days.

**Statistical analysis**

All the experiments were performed a minimum of three times. The results are expressed as the means ± S.E.M. and were analyzed via GraphPad Prism software (version 5). For comparisons between two groups, statistical significance was assessed via a two-tailed Student’s t-test. When more than two groups were compared, ANOVA was used to determine statistical significance. A p-value less than 0.05 was considered statistically significant.

**Supplementary Tables**

Table S1. The sequences of primers for qRT-PCR used in this study

| **Primer names** | **Forward(5’-3’)** | **Reverse(5’-3’)** |
| --- | --- | --- |
| **METTL3(Mouse)** | CAGTGCTACAGGATGACGGCTT | CCGTCCTAATGATGCGCTGCAG |
| **METTL14(Mouse)** | AGAGTGCGGATAGCATTGGTGC | CTCCTTCATCCAGACACTTCCG |
| **FTO(Mouse)** | GCCTCGGTTTAGTTCCACTCAC | GTCGCCATCGTCTGAGTCATTG |
| **ALKBH5(Mouse)** | TCGGAACCTGTGCTTTCTCTGC | CTTCCTGAGAATGATGACCGCC |
| **WTAP(Mouse)** | AGTGCCTGGAAGTTTACGCCTG | GCTTCAAGCTGTGCAATACGGC |
| **DNMT1(Mouse)** | GGACAAGGAGAATGCCATGAAGC | TTACTCCGTCCAGTGCCACCAA |
| **β-Actin(Mouse)** | CATTGCTGACAGGATGCAGAAGG | TGCTGGAAGGTGGACAGTGAGG |
| **METTL3(Human)** | CAAGGCTTCAACCAGGGTCT | GGTTTCCAAGGGTGATCCAGT |
| **DNMT1（human）** | AGGTGGAGAGTTATGACGAGGC | GGTAGAATGCCTGATGGTCTGC |
| **HNRNPA2B1(Human)** | ATTGATGGGAGAGTAGTTGAGCC | AATTCCGCCAACAAACAGCTT |
| **IGF2BP2(Human)** | AGCTAAGCGGGCATCAGTTTG | CCGCAGCGGGAAATCAATCT |
| **β-Actin(Human)** | TGGAACGGTGAAGGTGACAG | AACAACGCATCTCATATTTGGAA |

Table S2. The sequences of siRNAs used in this study

| **siRNAs names** | **Targeted sequences** |
| --- | --- |
| **si-h-METTL3[7]** | CAAGTATGTTCACTATGAA |
| **si-m-METTL3-001** | CAAGGAAGAGTGCATGAAA |
| **si-m-METTL3-002** | GAAAGGTCTTGGAGAGGTA |
| **si-m-METTL3-003** | TAAAGATTCATCAGTGTGC |
| **si-h-HNRNPA2B1-001** | GGAGAGTAGTTGAGCCAAA |
| **si-h-HNRNPA2B1-002** | AGCTGTTTGTTGGCGGAAT |
| **si-h-IGF2BP2[2]** | CAUGCCGCAUGAUUCUUGA |

**Reference：**

1. Li, F., C.A. Adase, and L.J. Zhang, *Isolation and Culture of Primary Mouse Keratinocytes from Neonatal and Adult Mouse Skin.* J Vis Exp, 2017(125).

2. Hu, X.T., et al., *The GRHL3-regulated long non-coding RNA lnc-DC modulates keratinocytes differentiation by interacting with IGF2BP2 and up-regulating ZNF750.* J Dermatol Sci, 2024. **113**(3): p. 93-102.

3. Hu, X.T., et al., *HDAC2 inhibits EMT-mediated cancer metastasis by downregulating the long noncoding RNA H19 in colorectal cancer.* J Exp Clin Cancer Res, 2020. **39**(1): p. 270.

4. Tang, Z., et al., *GEPIA: a web server for cancer and normal gene expression profiling and interactive analyses.* Nucleic Acids Res, 2017. **45**(W1): p. W98-W102.

5. Hainer, S.J. and T.G. Fazzio, *High-Resolution Chromatin Profiling Using CUT&RUN.* Curr Protoc Mol Biol, 2019. **126**(1): p. e85.

6. Wang, X.H., et al., *Fibroblast-like cells Promote Wound Healing via PD-L1-mediated Inflammation Resolution.* Int J Biol Sci, 2022. **18**(11): p. 4388-4399.

7. Wan, W., et al., *METTL3/IGF2BP3 axis inhibits tumor immune surveillance by upregulating N(6)-methyladenosine modification of PD-L1 mRNA in breast cancer.* Mol Cancer, 2022. **21**(1): p. 60.

**Supplementary results**


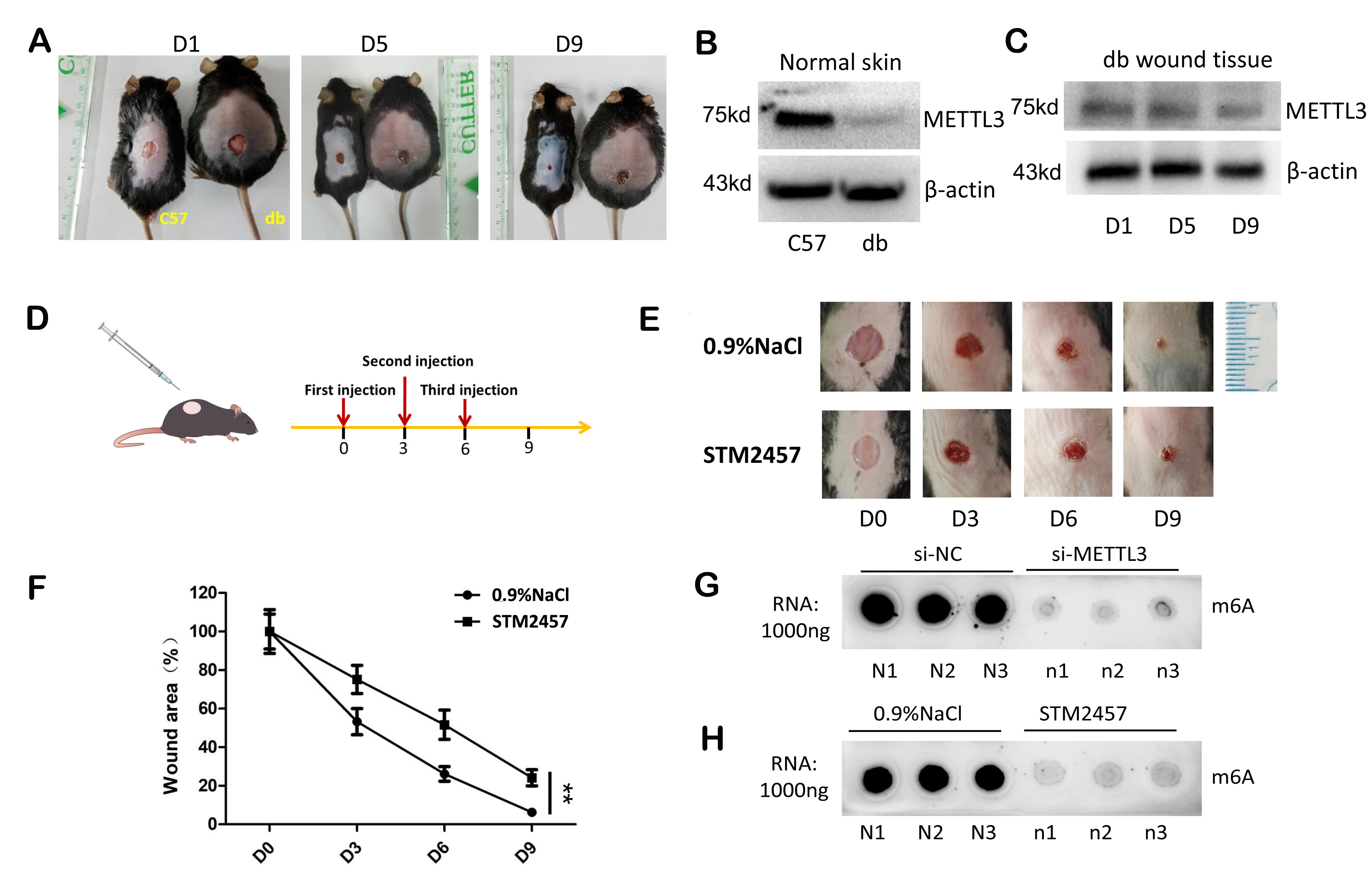


**Figure S1.** **METTL3 is a positive regulator of wound healing.** (A, B) METTL3 expression in the skin tissues of normal C57 BL/6J mice and diabetic mice detected by WB. (C) Changes in METTL3 expression during the wound healing process in diabetic mice detected by WB. (D-F) Effect of the METTL3 inhibitor STM2457 on mouse wound healing(n=5). STM2457(10µM) was injected into the wound site of the mice every three days. (G, H) Effects of METTL3 siRNAs(G) or STM2457(H) on m6A levels in wound site RNA analyzed by m6A dot blot. **P < 0.01; *P < 0.05.


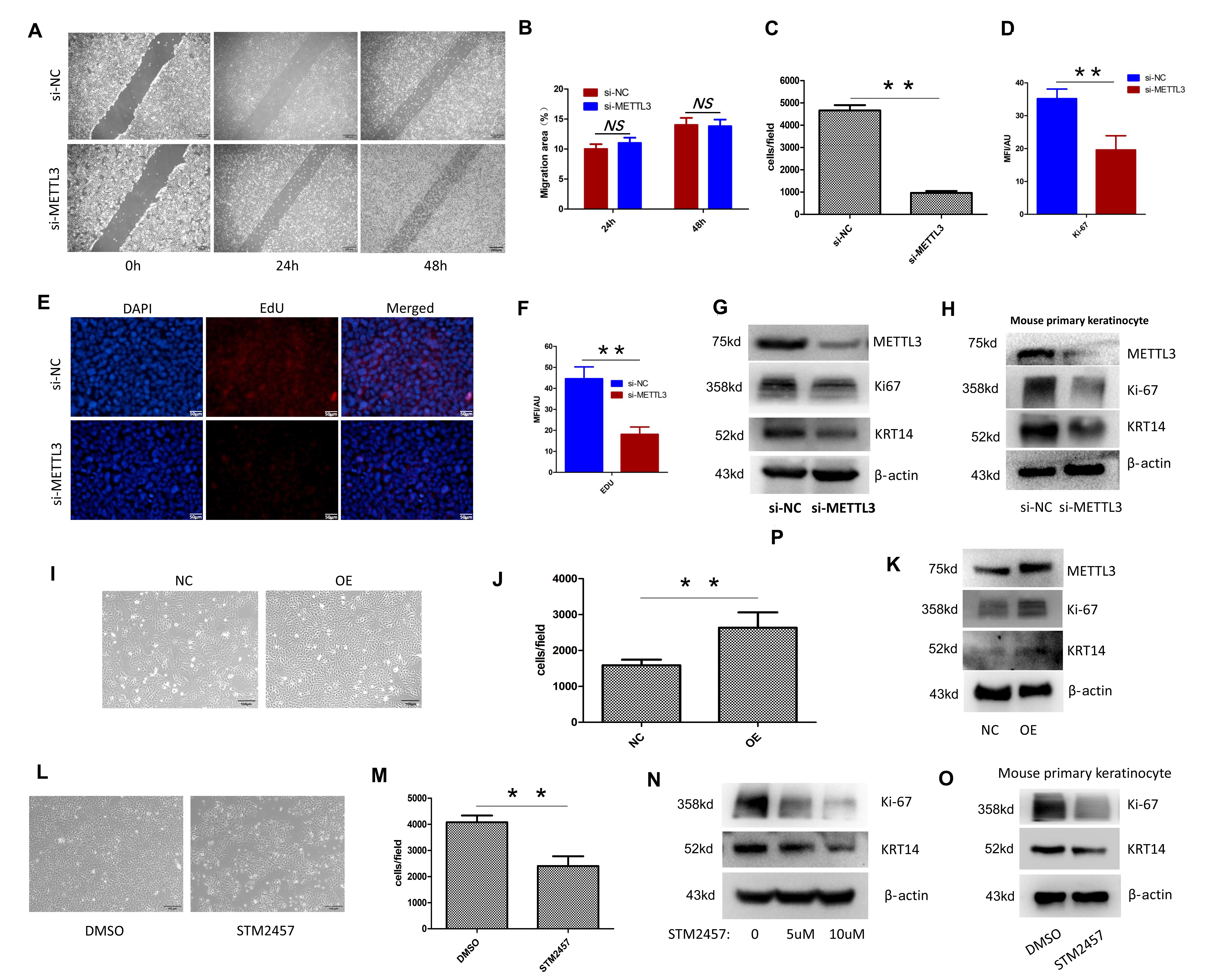


**Figure S2. METTL3 promotes keratinocytes proliferation.** (A, B) Evaluate the effects of METTL3 interference on the migration of HaCaT cells by wound healing assay. (C) Effects of METTL3 siRNAs on the growth quantity of HaCaT cells. (D) Effects of METTL3 siRNAs on the proliferation of HaCaT cells detected by Ki-67 staining. (E，F) Effects of METTL3 siRNAs on the proliferation of HaCaT cells detected by EdU staining. (G, H) The effects of METTL3 interference on the expression of proliferation markers in HaCaT cells(G) and primary keratinocytes(H) analyzed by WB. (I-K) The impact of METTL3 overexpression on the growth of HaCaT cells(I,J) and the expression of proliferation markers(K). (L, M) The effect of the METTL3 inhibitor STM2457 (10µM for 48h) on the growth of HaCaT keratinocytes. (N) Effects of STM2457 on the expression of proliferation markers in keratinocytes detected by WB. (O) Effects of STM2457 on the expression of proliferation markers in primary keratinocytes detected by WB. NS: no significance；**P < 0.01; *P < 0.05.


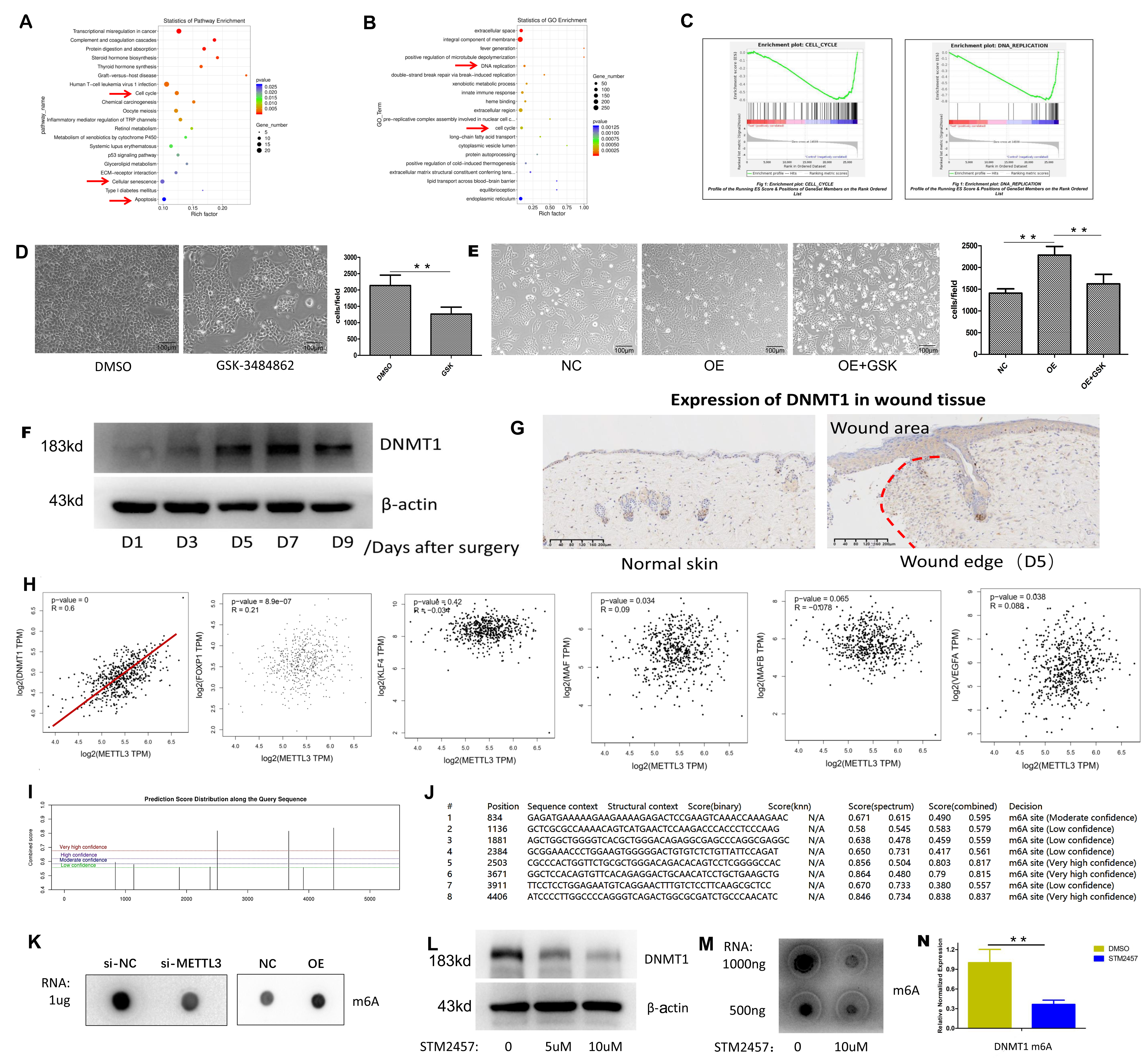


**Figure S3. DNMT1 is a downstream target gene of METTL3 that regulates keratinocyte proliferation.** (A, B) KEGG(A) and GO(B) enrichment analyses of the EDGs. (C) GSEA analysis of the effects of METTL3 interference on cell cycle and DNA replication. (D) Effects of the DNMT1 inhibitor GSK-3484862 on the growth of HaCaT cells. HaCaT cells were treated with 10µM for GSK-3484862 for 48h. (E) Effects of the DNMT1 inhibitor GSK-3484862 on the growth of METTL3 over-expressed HaCaT cells. (F) Changes in DNMT1 expression during the wound healing process detected by WB. (G) Immunohistochemical staining for METTL3 expression at the wound site (day 5). (H) Analysis of the correlation of METTL3 expression with that of genes such as DNMT1 in skin tissues. The data were collected from the GTEx database and analyzed by GEPIA (<http://gepia.cancer-pku.cn/>). (I, J) Prediction of m6A methylation sites in DNMT1 mRNA by SRAMP (SRAMP: http://www.cuilab.cn/sramp). (K) Effects of METTL3 interference and overexpression on overall RNA m6A levels analyzed by m6A dot blot. (L-N) Effects of the METTL3 inhibitor STM2457 (5-10 µM for 48 h) on DNMT1 protein expression(L) and m6A levels in mRNA (M, N). **P < 0.01; *P < 0.05.


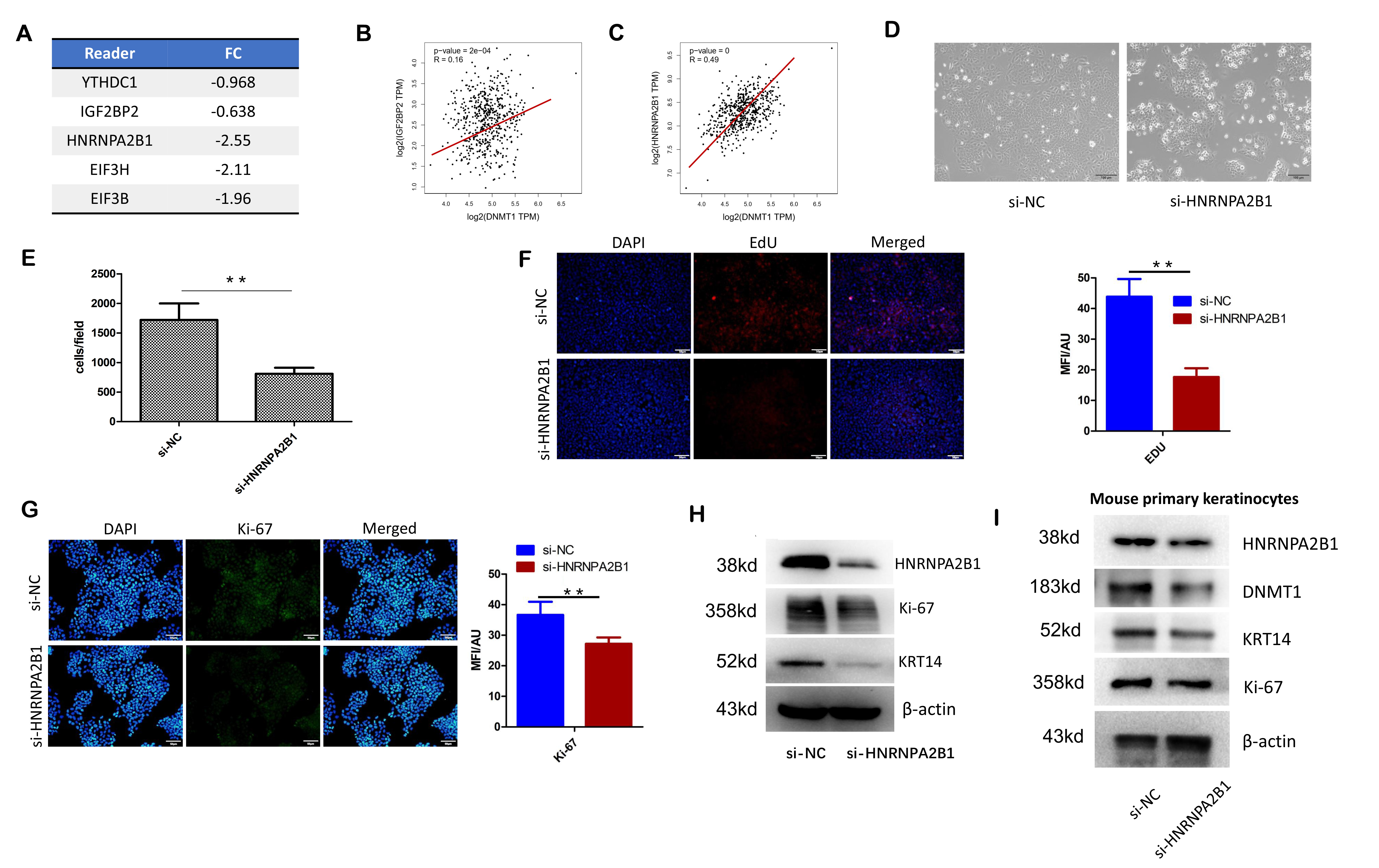


**Figure S4.** **HNRNPA2B1 is an m6A reader that regulates the expression of DNMT1.** (A) Candidate m6A "readers" and their fold changes. Data were collected from GSE18590. (B, C) Analysis of the correlation between IGF2BP2(B) or HNRNPA2B1(C) expression and DNMT1 in skin tissue. (D, E) Interfering with HNRNPA2B1 inhibited the growth of HaCaT cells. (F, G) Effects of interfering with HNRNPA2B1 on the proliferation of HaCaT cells detected by EdU(F) and Ki-67 staining(G). (H, I) WB analysis of the effects of HNRNPA2B1 interference on the expression of DNMT1 and proliferation marker in HaCaT cells(H) and primary mouse keratinocytes(I). **P < 0.01; *P < 0.05.


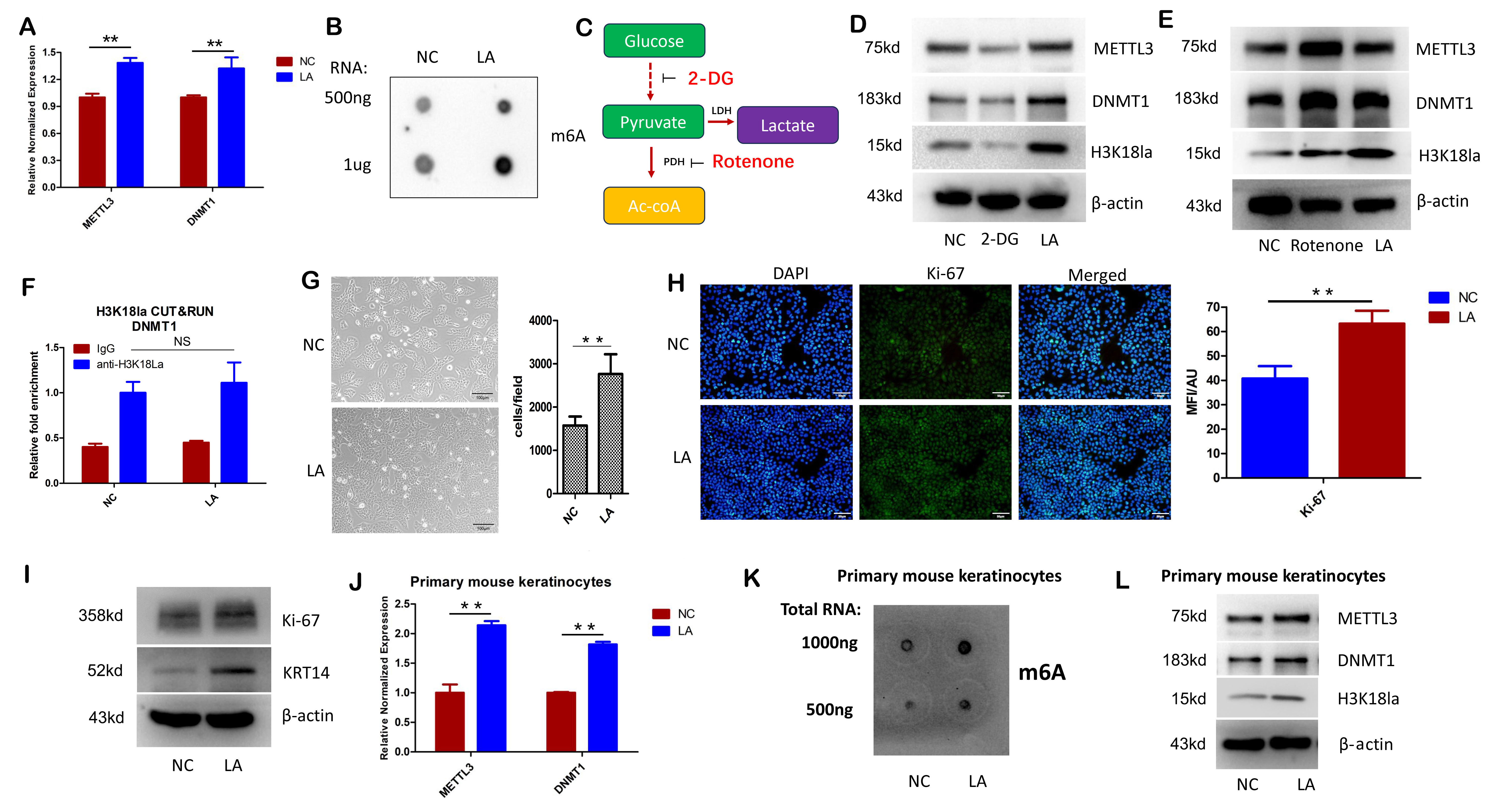
**Figure S5. Lactate regulates the expression of METTL3 through inducing histone lactylation.** (A) Effect of lactate stimulation on the transcriptional levels of METTL3 and DNMT1 detected by qPCR. (B) The effect of lactate treatment on overall m6A levels in HaCaT cells RNA analyzed by m6A dot blot. (C-E) The effect of interfering with intracellular lactate synthesis on H3K18la, METTL3, and DNMT1 expression detected by WB. (F) Effects of lactate treatment on the binding of H3K18la to the DNMT1 promoter detected by the CUT&RUN assay. (G) The effect of lactate treatment on the growth of HaCaT cells. (H)Effects of lactate stimulation on the proliferation of HaCaT cells detected by Ki-67 staining. (I) Effects of lactate treatment on the expression of proliferation markers in HaCaT cells detected by WB. (J) qPCR analysis of the effects of lactate treatment on DNMT1 expression in primary mouse keratinocytes. Cells were treated with 25mM lactate for 24h.(K) m6A dot blot analysis of the effects of lactate treatment on RNA m6A levels in primary mouse keratinocytes. (L) WB analysis of the effects of lactate treatment on the expression of DNMT1 and proliferation markers in primary mouse keratinocytes.NC: negative control; LA: lactate. NS: no significance；**P < 0.01; *P < 0.05.


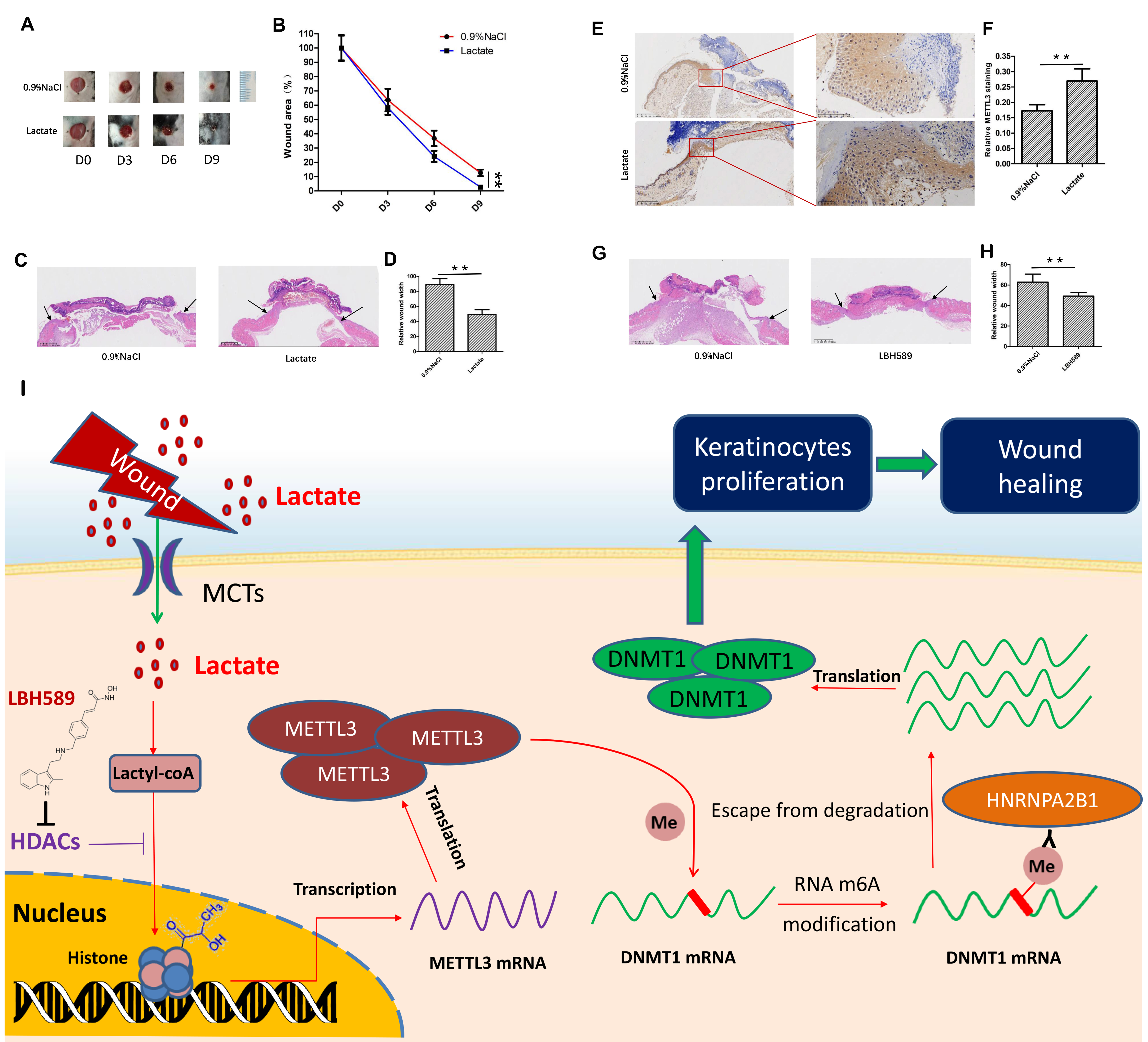


**Figure S6. Lactate and HDACs inhibitor LBH589 promote METTL3 expression and wound healing.** (A, B) Effect of lactate on wound healing in mice(n=5). 25mM lactate was locally injected into the wound site of the mice every three days. (C, D) Effects of lactate on the width of wounds in mice. (E, F) Effect of lactate on METTL3 expression in the wound tissue of mice detected by IHC. (G, H) Effects of LBH589 on the width of wounds in mice. (I) Schematic diagram illustrating the molecular mechanism by which lactylation-driven METTL3 promotes wound healing. **P < 0.01; *P < 0.05.
